# Influence of ankle joint position on angles and distances of the ankle mortise using intraoperative cone beam CT: A cadaveric study

**DOI:** 10.1371/journal.pone.0217737

**Published:** 2019-05-31

**Authors:** Sven Y. Vetter, Maxim Privalov, Nils Beisemann, Benedict Swartman, Holger Keil, Joachim Kirsch, Paul Alfred Grützner, Jochen Franke

**Affiliations:** 1 BG Trauma Center Ludwigshafen at Heidelberg University Hospital, Ludwigshafen, Germany; 2 Institute for Anatomy and Cell Biology, University of Heidelberg, Heidelberg, Germany; Public Library of Science, UNITED KINGDOM

## Abstract

**Background:**

The precise anatomical reduction of the ankle mortise is crucial for the clinical outcome in unstable syndesmotic injuries. Intraoperative cone beam computed tomography (CT), in addition to two-dimensional fluoroscopy, provides detailed information about the reduction and implant placement. The aim of this study was to analyze the influence of the joint position on the fibula position in the incisural notch and to determine the inter- and intraindividual anatomical differences in the intact ankle joints.

**Methods:**

A total of 20 fresh-frozen lower legs disarticulated in the knee joint of 10 individuals were included. The measurements were performed using a cone beam CT. The distances and angles were measured in the standard imaging planes. The mean values of distances and angles were compared during the different joint positions: 10° dorsiflexion, 0° neutral position and 20° plantar flexion.

**Results:**

The influence of the joint position was on average as follows: The anterior tibiofibular distance was 3.68 mm in 10° dorsiflexion, 3.66 mm (0° neutral position) and 3.59 mm (20° plantar flexion). The posterior tibiofibular distance measured 7.82mm, 7.76mm and 7.82mm. The rotation of the fibula measured ten millimeters proximal the joint line was 1.2°, 1.3° and 1.05°. The fibular rotation determined 4mm was 9.3°, 9.4° and 9.4°. On average, the following intraindividual variations were observed: superior tibiotalar clear space of 0.27mm and 0.15mm medial; and anterior tibiofibular distance of 0.42mm, 0.38mm posterior and 0.24mm in the incisural notch. The proximal angle of the fibular rotation was 0.2° and distal 0.4°. The interindividual variations of the angles and distances exceeded the intraindividual values partly by 3 to 4 fold.

**Conclusions:**

Within the scope of this study neither the tibiofibular distance, nor the tibiofibular angle changed significantly through the different joint positions. The intraindividual differences were little while the interindividual variations of the parameters were distinctive.

## Introduction

The fracture of the ankle accounts for 9% of all fractures of the human skeletal system. Unstable syndesmotic injuries occur in up to every seventh ankle fracture [1]. The high socioeconomic relevance of this injury has already been proven in the context of retrospective studies [2, 3].

The surgical treatment pursues the objective of an anatomical reduction of the syndesmotic area [4–8]. If there is insufficient anatomical reconstruction, like incongruity of the articular surfaces or remaining gaps of the fracture, a premature chondral degeneration with osteoarthritis can be expected [6, 9, 10]. Biomechanical and clinical studies have shown that a shortening of the fibula by more than 2 mm or rather its increased external rotation in the incisural notch by more than 5° results in a distinctive superior stress on the involved joint surfaces [9]. At the same time other anatomical studies have proven that the interindividual width of the syndesmosis has no significant differences [11].

The knowledge and the information about the normal anatomical position and the initial surgical situation as well as the result in the process of fracture reduction are crucial.

The visualization of the ankle and its involved structures using conventional two-dimensional fluoroscopy is challenging [12, 13]. The intraoperative use of three-dimensional imaging in the treatment of the syndesmotic injuries provides additional information for the detection of malalignment. [6, 14, 15].

Depending on the literature examined it becomes apparent that in 7.3% to 43% of the studied cases of different intraarticular fractures a therapeutic consequence for the patient's treatment due to the information obtained by using intraoperative three-dimensional imaging was observed[16–22]. It has already been shown, in the context of investigations on the calcaneal offsets, that measurements performed with data, acquired by weight bearing radiographs and 3D reconstructions, provide increased accuracy in comparison to information gained by 2D imaging and are therefore relevant for surgical planning [23]. Especially in the case of acute unstable syndesmotic injuries the three-dimensional scan altered the surgical procedure in 30% of all treatments [24–26].

The position in which the investigation takes place has yet not been analyzed.

The aim of our study was to evaluate the influence of the ankle joint position on the rotation and position of the fibula in the fibular notch of the tibia. Additionally the intra (left and right)—and interindividual differences in the anatomical parameters of the ankle mortise utilizing three-dimensional imaging were supposed to be determined.

The three different positions of the ankle joint with 20° plantar flexion, 0° neutral position and 10° dorsiflexion were chosen to simulate the most frequent utilized positions and to evaluate the superior position of the ankle joint during an intraoperative cone beam computed tomography (CT) scan.

## Material and methods

### Cadaveric model

Intact fresh-frozen lower legs were included in this experimental study which were disarticulated in the knee joint. Excluded were individuals with prior injuries, severe osteoarthritis, deformities or with osteosynthesis of the lower legs. The measurements were performed using a cone beam CT (Arcadis Orbic 2, Siemens, Erlangen, Germany). The image data sets were evaluated in the standard imaging planes. The distances between the anterior and posterior edges of the tibia and the fibula, and the angle of the fibula to the tibia 10 mm proximal the tibial articular surface and 4 mm distal the talar articular surface were measured in the axial view. Since there is no established method for the determination of the fibular rotation, we applied two different techniques to avoid measurement errors. In the context of the anatomical comparison the tibiofibular distance in the fibular notch, and the tibiotalar distances, the superior and medial clear space, were additionally determined in the 0°-neutral position using the coronal plane.

### Experimental setting

**Fig 1:**
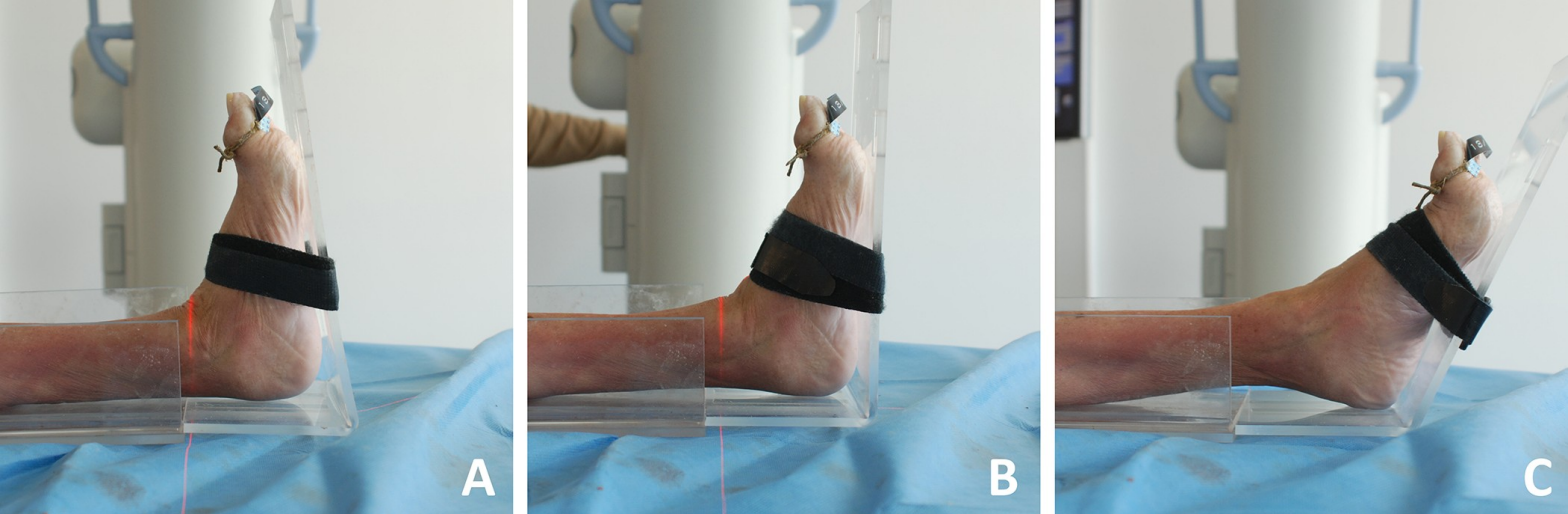
**Photographs of the set before the 3D-scan of the intact ankle in 10° dorsiflexion (A), in 0° neutral position (B) and in 20° plantar flexion (C)**.

The experimental setting was designed to imitate the real surgical conditions. A mobile and height-adjustable radiolucent carbon fiber table served as a working station and positioning area for the fresh-frozen lower legs. The self-designed holding device for the cadaveric lower legs was made of acrylic glass, not only because it is a particularly flexible and plastic material but also because it provides, as well as the carbon itself, artifact-free visualization in radiological imaging. The immobilization was performed by commercially available Velcro strips, which are also radiolucent. The foot portion of the holding device, in which the ankle is positioned, was designed to be removable and exchangeable by other portions with variable properties. A total of three different foot portions were manufactured to simulate: 10° dorsiflexion, 0° neutral position and 20° plantar flexion (Fig 1). This construction enabled us to change the ankle position in a controlled manner and to make this situation reproducible as well.

### Visualization software: Syngo

The radiological analysis is achieved by using the visualization software “syngo” (Siemens, Erlangen, Germany). This is a DICOM viewer that visualizes the two-dimensional data set as well as the multiplanar reconstructions of the three-dimensional data set. Moreover, the implemented tools in this software can be used for measuring distances and angles. What it looks like in detail, will be explained in the following section and clarified with the aid of an example.

### Acquisition of data: Step by step

**Fig 2:**
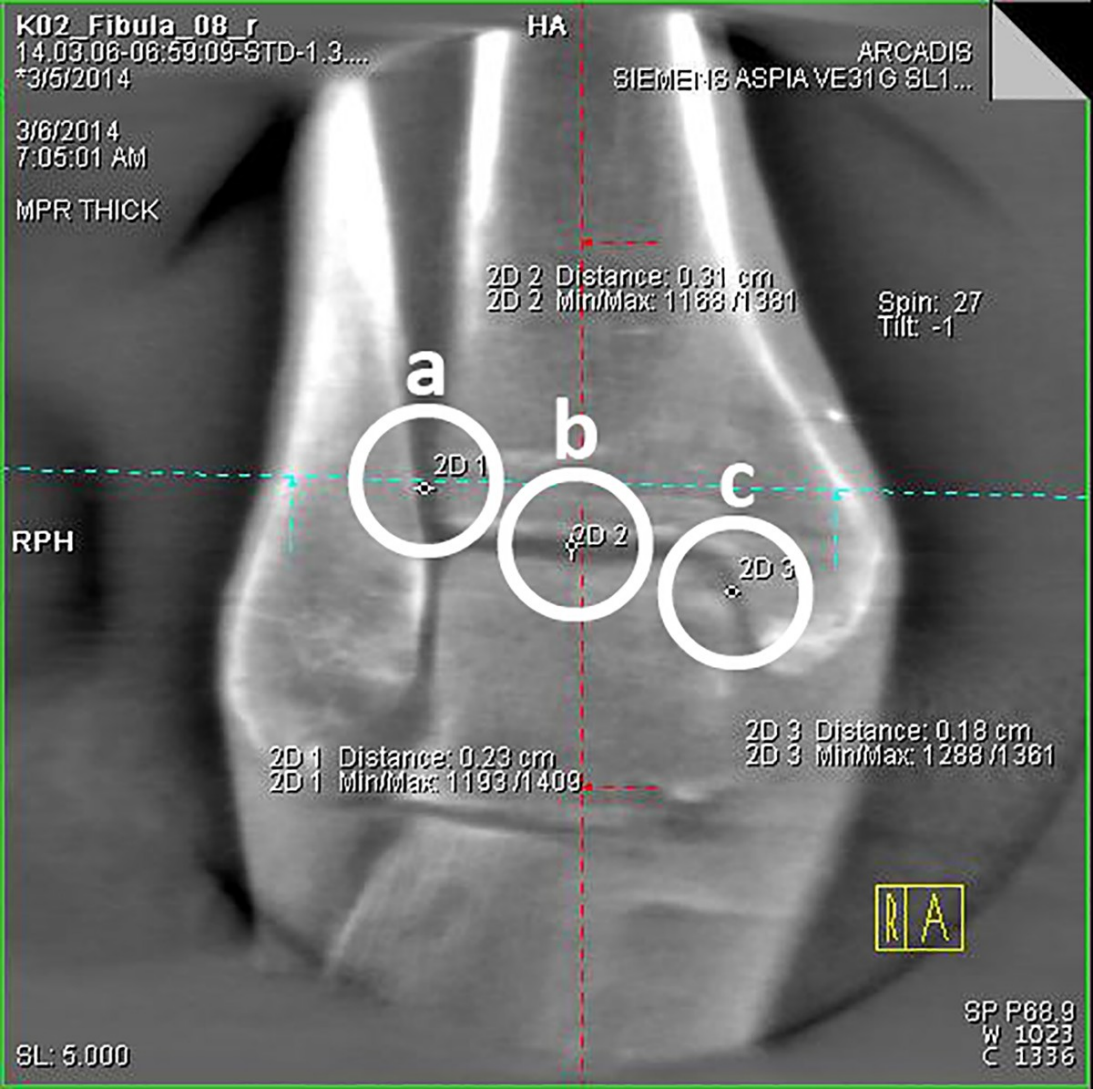
**3D-reconstruction, coronal plane: determination of the tibiofibular distance (a), the superior tibiotalar clear space in the center of the ankle joint (b) and the medial tibiotalar clear space (c)**.

**Fig 3:**
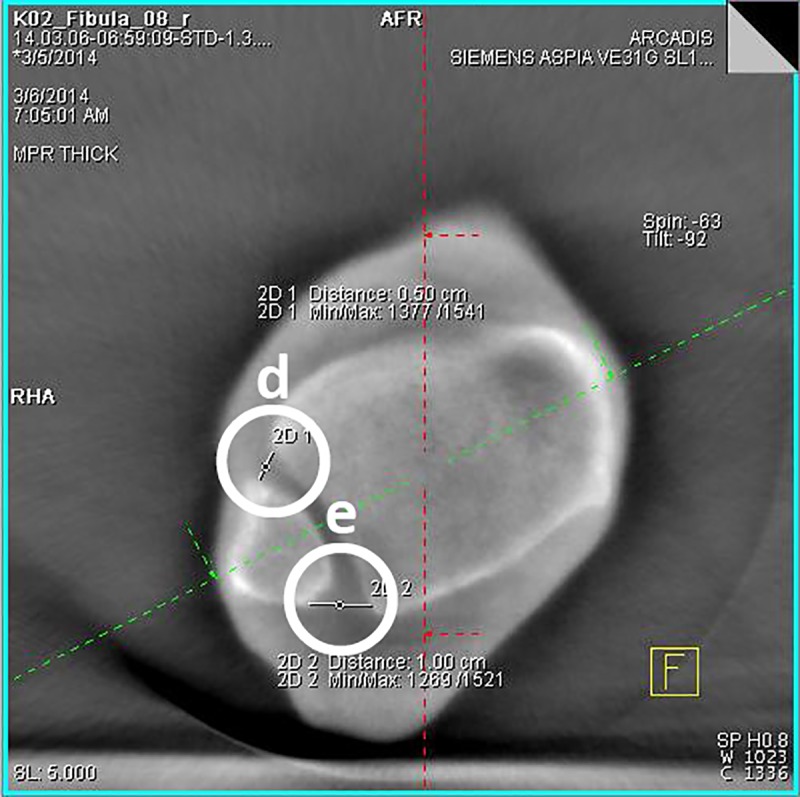
**3D-reconstruction, transversal plane: determination of the anterior (d) and the posterior tibiofibular distance (e)**.

**Fig 4:**
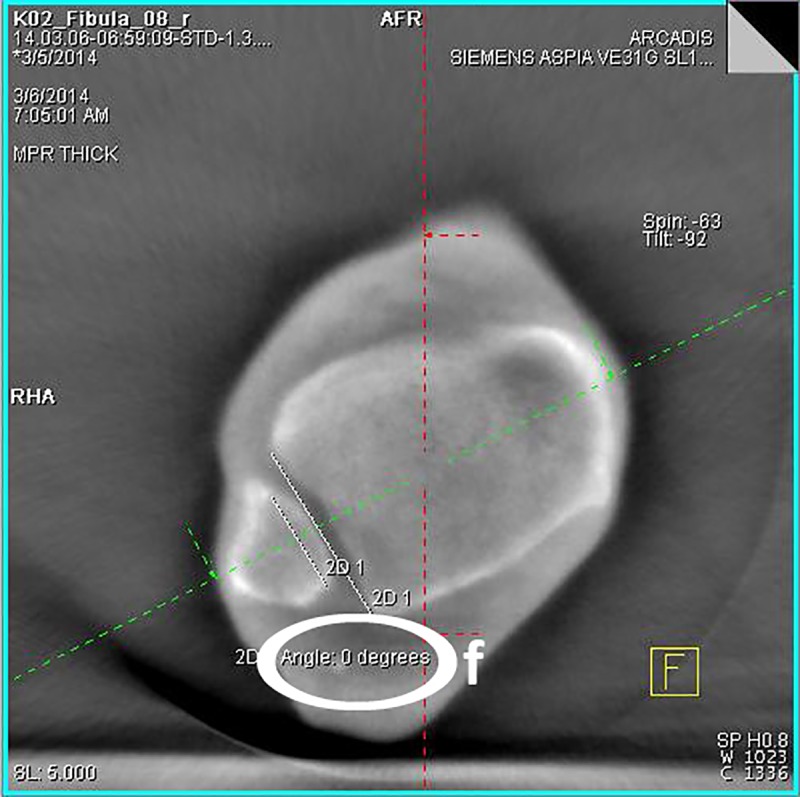
**3D-reconstruction, transversal plane: determination of the fibular rotation angle 10 mm above the tibial articular surface (f)**.

**Fig 5:**
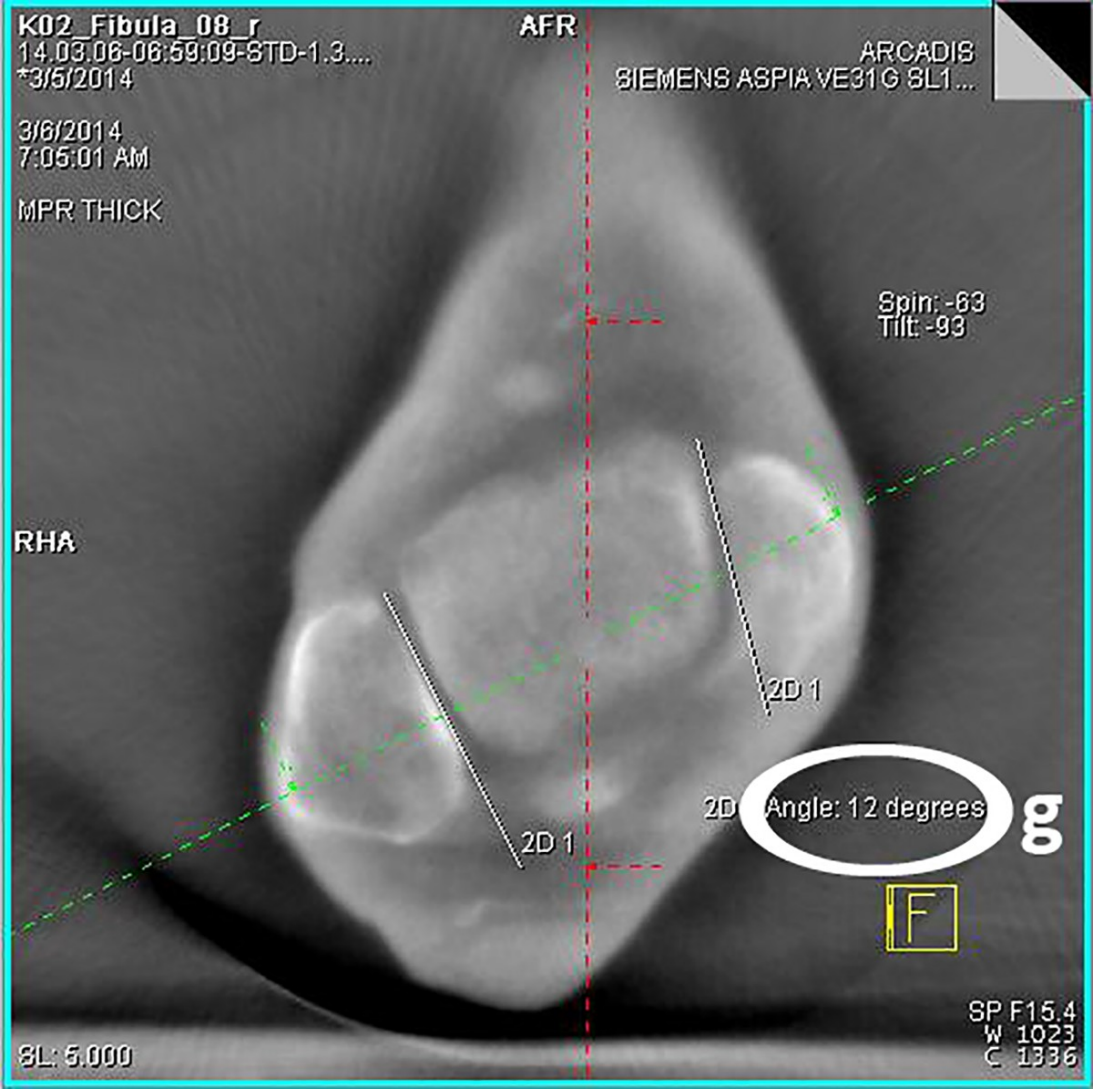
**3D-reconstruction, transversal plane: determination of the rotation angle of the fibula 4 mm below the talar articular surface (g)**.

1. Step: After loading the image data set, obtained during the experimental series, the ankle joint has to be positioned in the frame center of each imaging plane according to the radiological standard planes of the ankle joint in the computed tomography.
2. Step: The adjustment of the coronary axis is performed in the sagittal plane, making it orthogonal to the tibial articular surface and running through the center of the ankle joint. The adjustment of the transversal axis is also realized in the sagittal plane, so that it is positioned perpendicularly to the coronal axis at a measured height of 10 mm above the tibial articular surface.
3. Step (Fig 2): In this now adjusted coronal plane the values for the tibio-fibular distance are determined in the incisural notch (a) at the level of the transversal axis, 10 mm above the tibial articular surface.
4. Step (Fig 2): The determination of the superior tibiotalar clear space in the center of the ankle joint (b), as well as the medial tibiotalar clear space (c), is performed in the coronal plane.
5. Step (Fig 3): In the viewing field of the transversal plane the anterior (d) and the posterior tibiofibular distance (e) are measured by connecting the edges of the cortical bone.
6. Step (Fig 4): The measurement of the fibular rotation angle 10 mm above the tibial articular surface (f) is carried out at the already established adjustments of the axes in the transversal plane, wherein an angle between the sagittal axis of the tibial incisural notch and the sagittal axis of the fibula is formed.
7. Step: Prior to the determination of the fibular rotation below the talar articular surface, the transversal axis has to be adjusted in the coronal plane, so that it becomes positioned 4 mm below the talar articular surface.
8. Step (Fig 5): In a final step, the rotation angle of the fibula 4 mm below the talar articular surface (g) can now be quantified in the transversal plane. Here, an angle between a straight line, adjacent to the medial surface of the lateral malleolus, and another, adjacent to the lateral surface of the medial malleolus, is formed.

### Statistical analysis

Means and standard deviations were calculated and paired t-tests were performed to answer the question about how the angle and distance measurements behave in relation to the procedures carried out during the test series. The mean values of distances and angles in the intact ankle were compared in different joint positions: 10° dorsiflexion, 0° neutral position and 20° plantar flexion. The statistical analysis was performed using SPSS (IBM SPSS Statistics, Version 21.0.0.2) on the basis of the tabularly acquired dataset using EXCEL (Microsoft Excel 2013, Version 15.0.4779.1001). The significance threshold was set at p ≤ 0.05.

The cadavers used in this study were received from the Institute for Anatomy and Cell Biology, University of Heidelberg, Germany. The deceased provided written informed consent for the use of their body for research purposes. The study was approved by the ethics committee of the Medical Faculty of Heidelberg. The application was submitted on 13.01.2014 and was accepted on 17.02.2014 with the registration number S-013/2014.

## Results

The fresh-frozen lower legs of 4 male and 6 female human cadaver specimens were studied. The average age of the individuals included was 83.8 years.

The mean values of the distances and angles of the mortise depending on the ankle joint position are displayed in Table 1.

**Table 1:** Distances and angles of the mortise depending on ankle position.

anatomical parameter	10° dorsiflexion	0° neutral position	20° plantar flexion	Sig. (2-sided)
TFD (anterior)	3.68 ± 1.07 mm	3.66 ± 1.05 mm	3.6 ± 1.07 mm	0.530; 0.091; 0.169
TFD (posterior)	7.83 ± 1.24 mm	7.76 ± 1.18 mm	7.82 ± 1.14 mm	0.352; 0.905; 0.304
AFR (10 mm proximal)	1.2°± 1.28	1.2°± 1.28	1.05°± 1.1	1.0; 0.083; 0.083
AFR (4 mm distal)	9.3°± 2.81	9.4°± 2.85	9.4°± 2.87	0.163; 0.330; 1.0

Table 1: Mean values and standard deviations of the distances and angles depending on the ankle joint position. 2-sided significance level of the paired sample t-tests. TFD = tibiofibular distance; AFR = angle of fibular rotation; sig. = significance level (alpha)

### Intraindividual comparison

**Fig 6:**
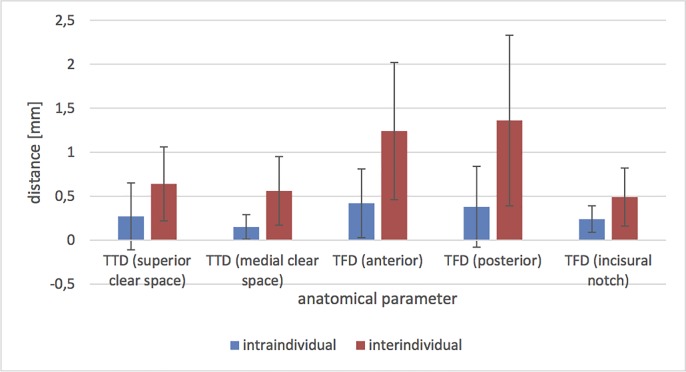
Intra- and interindividual mean values and standard deviations of the differences of the distances in the 0° neutral position. TTD = tibiotalar distance; TFD = tibiofibular distance.

**Fig 7:**
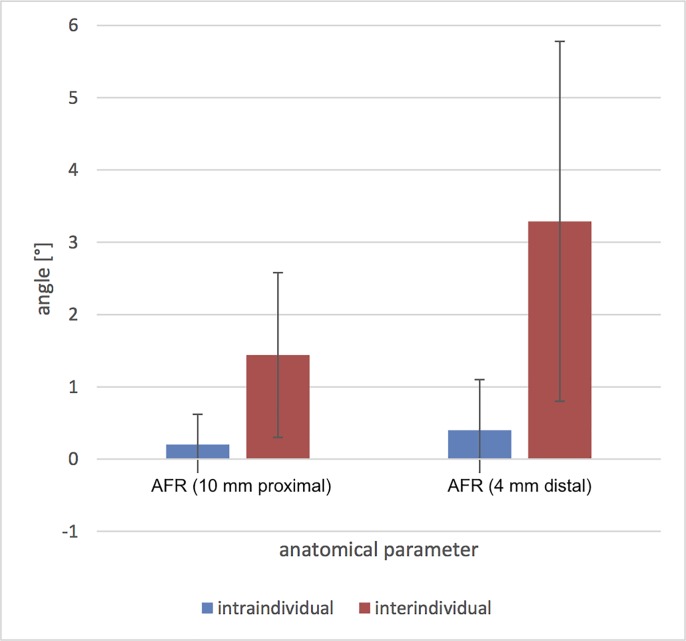
Intra- and interindividual mean values and standard deviations of the differences of the angles in the 0° neutral position. AFR = angle of fibular rotation.

On average, the following intraindividual (left vs. right) variations were observed (Figs 6 and 7): superior tibiotalar clear space of 0.27 ± 0.38 mm and 0.15 ± 0.14 mm medial; and anterior tibiofibular distance of 0.42 ± 0.39 mm, 0.38 ± 0.46 mm posterior and 0.24 ± 0.15 mm in the fibular notch. The angle of the fibular rotation 10 mm proximal to the tibial articular surface was 0.2° ± 0.42 and 4 mm distal to the talar articular surface 0.4° ± 0.7.

### Interindividual comparison

The interindividual differences in the distances were (Figs 6 and 7): superior tibiotalar clear space 0.64 ± 0.42 mm and 0.56 ± 0.39 mm medial; and anterior tibiofibular distance 1.24 ± 0.78 mm, 1.36 ± 0.97 mm posterior and 0.49 ± 0.33 mm in the fibular notch. The angle of the fibular rotation 10 mm proximal to the tibial articular surface was 1.44° ± 1.14 and 4 mm distal to the talar articular surface 3.29° ± 2.49.

## Discussion

### Ankle joint position and fibula position in the incisural notch

The main findings of the study were that neither the tibiofibular distance nor the tibiofibular angle changed significantly through the different joint positions. Considering the images from cone beam CT, there were no significant differences of the values associated with the position of the ankle in 10° dorsiflexion, 0° neutral position and 20° plantar flexion detectable. This aspect has a high clinical relevance when applying intraoperative cone beam CT since there existed uncertainty about the optimal ankle position during the cone beam CT scan.

These findings are in contrast to the results of Nault et al. in an MRI-study of intact non-weight bearing ankle joints [27]. The published results show significant differences of tibiofibular distances and fibula rotation depending on dorsi- and plantarflexion. It hast to be emphasized that accurate ankle positions were not explicitly stated in the article. In fact the published image demonstrates a plantarflexion of at least 40 degrees. Additionally a precise location of the measurements in the axial views was not defined. These results together illustrate the necessity of clear measurement methods to gain comparable data.

### Anatomical comparison

Our study reveals that the intraindividual differences were small while the interindividual variations of certain parameters of the ankle were distinctive. These results indicate relevant differences of anatomical parameters of the ankle between individuals. Another notable finding is that, in spite of numerous matches, the distances and angles related to the fibular rotation differ little. However, in the interindividual comparison it has to be pointed out that the variations of the angles and distances considerably exceed the intraindividual values.

Significantly larger interindividual—compared to the intraindividual differences were already reported in anatomical studies of the proximal and distal femur as well as the joint surfaces of the calcaneus [28, 29]. Especially the shape of the fibula and the cavity of the fibular notch vary significantly between individuals [30] [31].

Therefore, in the context of the surgical treatment, preoperatively acquired CT scans, if available, should be taken into account, or otherwise the relevant anatomical parameters of the contralateral intact ankle can also be obtained intraoperatively by using cone beam CT. This additional information provides a better pre- and intraoperative planning and therefore can improve the intraoperative treatment in complex ankle fractures.

The main limitations of this study are the small number of cases studied, the study set-up with fresh frozen lower legs and the age of individuals included. Indeed there was no indication that degenerative alterations influenced the data. The specimens were analyzed for previous injury, surgery, osteoarthritis, and anatomic aberrations.

The performed injury in the specimen model was restricted solely to the syndesmotic complex leaving the fibula intact. It is well known that syndesmotic injuries are often accompanied by fibula fractures. An additional osteotomy of the fibula simulating a fracture which is reduced and exactly anatomically fixed with a plate presumably does not influence distances of the ankle mortise in a non-weight bearing specimen model.

Furthermore, it should be noted that the intraoperative cone beam CT, as it is not a weight bearing device, is a limitation with respect to the effects of weight and also the actions of muscles and fascias. In addition the amount of radiation, cost and size of the intraoperative cone beam CT has to be taken into account when using in the clincial routine.

There exists a potential for errors in the surgical procedure and data collection. However, the experiments were carried out as a single observer (M.P.) study under the direct instruction of an experienced senior foot and ankle orthopaedic surgeon (S.Y.V) to minimize any confounding observer variability. A reproducible and accurately described method was applied. We believe that the results thoroughly represent the quantitative anatomic characteristics of the ankle mortise after syndesmotic injury.

Other soft tissue around the ankle joint like the achilles tendon tension did not seem to play a significant role. In all individuals the pursued plantar- and dorsiflexion could be achieved without significant force.

In summary, the key findings are that the anatomical parameters of the ankle joint in the intraindividual comparison of the left and right side are comparable, whilst the interindividual variations of certain parameters were distinctive, and that the joint position of the intact ankle has no significant effect on the position of the fibula in the fibular notch. Both of these findings are useful for obtaining an exact anatomical reconstruction in unstable syndesmotic injuries using intraoperative cone beam CT. A special taping of the foot or ankle during the intraoperative cone beam CT scan to obtain a certain ankle position appears to be negligible. Additionally, this study demonstrates the individual similarity of the ankle mortise which leads to the conclusion that a preoperative CT scan or a cone beam CT scan of the uninjured side to obtain an anatomical ankle blueprint has to be considered. At this point it should be taken into account that cone beam CT provides intraoperative imaging which can be used to assess distances and angles of the ankle mortise to accurately analyze fracture reduction and implant placement [32].

Accordingly, within the scope of the surgical treatment it can be concluded that for the reduction of the syndesmotic complex the measured angles and distances of the intact ankle should be considered.

## Supporting information

S1 Table Distances and angles of the ankle mortise. (XLSX)
